# Supplementary material for: Evolution of world running record performances for men and women: physiological characteristics
Source: Front Physiol. 2024 Mar 13;15:1372092. doi: 10.3389/fphys.2024.1372092 (PMC10965763; doi:10.3389/fphys.2024.1372092)
Supplement: Supplementary file 1 [file DataSheet1.PDF]

Supplementary Material

Below we provide tables for the analyzed WR data for men and women. For each year, the tables give the fitted model parameters, and for each distance the actual WR times (1st line), the model estimated times (2nd line), and the relative error in percent (3rd line). Also given is the average error for all distances in percent, and the WRs and predicted times for the half and full marathon.

Magenta color indicates a new WR in the given year. Red (green) color marks the slowest (fastest) time relative to the model fit.

World Records Men

Year	t_c	v_m [m/min]	E_s	E_l	γ_{an}	γ_{ae}	1000	1500	1609.34	2000	3000	5000	10 000	av.err [%]	Halfmarathon	Marathon
1918	06:26.51	358.27	0.48	5.06	0.135	0.062	02:29.10<br>02:28.34<br>−0.51	03:54.70<br>03:55.49<br>0.34	04:12.60<br>04:15.25<br>1.05	05:30.40<br>05:27.65<br>−0.83	08:33.20<br>08:31.23<br>−0.38	14:36.60<br>14:42.28<br>0.65	30:58.80<br>30:54.09<br>−0.25	0.57	1:08:58.72	2:36:06.60<br>2:25:51.08<br>−6.57
1919	06:26.51	358.27	0.48	5.06	0.135	0.062	02:29.10<br>02:28.34<br>−0.51	03:54.70<br>03:55.49<br>0.34	04:12.60<br>04:15.25<br>1.05	05:30.40<br>05:27.65<br>−0.83	08:33.20<br>08:31.23<br>−0.38	14:36.60<br>14:42.28<br>0.65	30:58.80<br>30:54.09<br>−0.25	0.57	1:08:58.72	2:36:06.60<br>2:25:51.08<br>−6.57
1920	06:26.51	358.27	0.48	5.06	0.135	0.062	02:29.10<br>02:28.34<br>−0.51	03:54.70<br>03:55.49<br>0.34	04:12.60<br>04:15.25<br>1.05	05:30.40<br>05:27.65<br>−0.83	08:33.20<br>08:31.23<br>−0.38	14:36.60<br>14:42.28<br>0.65	30:58.80<br>30:54.09<br>−0.25	0.57	1:08:58.72	2:32:35.80<br>2:25:51.08<br>−4.42
1921	06:43.68	356.18	0.48	6.18	0.135	0.055	02:29.10<br>02:28.34<br>−0.51	03:54.70<br>03:55.49<br>0.34	04:12.60<br>04:15.25<br>1.05	05:30.40<br>05:27.65<br>−0.83	08:33.20<br>08:32.05<br>−0.22	14:36.60<br>14:39.92<br>0.38	30:40.20<br>30:37.42<br>−0.15	0.50	1:07:50.35	2:32:35.80<br>2:22:18.41<br>−6.74
1922	06:42.91	359.20	0.45	5.22	0.126	0.061	02:28.60<br>02:28.35<br>−0.17	03:54.70<br>03:54.56<br>−0.06	04:12.60<br>04:14.05<br>0.57	05:26.40<br>05:25.30<br>−0.34	08:28.60<br>08:28.26<br>−0.07	14:35.40<br>14:36.39<br>0.11	30:40.20<br>30:39.36<br>−0.05	0.20	1:08:19.47	2:32:35.80<br>2:24:14.00<br>−5.48
1923	07:00.96	358.25	0.45	5.20	0.125	0.061	02:28.60<br>02:28.15<br>−0.30	03:54.70<br>03:54.06<br>−0.27	04:10.40<br>04:13.47<br>1.23	05:26.40<br>05:24.40<br>−0.61	08:28.60<br>08:28.26<br>−0.07	14:35.40<br>14:36.39<br>0.11	30:40.20<br>30:39.36<br>−0.05	0.38	1:08:19.47	2:32:35.80<br>2:24:14.00<br>−5.48
1924	07:24.19	356.56	0.45	7.92	0.126	0.048	02:28.60<br>02:27.82<br>−0.52	03:52.60<br>03:53.53<br>0.40	04:10.40<br>04:12.90<br>1.00	05:26.40<br>05:23.66<br>−0.84	08:28.60<br>08:28.12<br>−0.09	14:28.20<br>14:29.59<br>0.16	30:06.20<br>30:05.03<br>−0.07	0.44	1:06:10.40	2:32:35.80<br>2:17:48.63<br>−9.69
1925	07:21.52	357.25	0.45	7.52	0.124	0.050	02:28.60<br>02:27.87<br>−0.49	03:52.60<br>03:53.46<br>0.37	04:10.40<br>04:12.79<br>0.95	05:26.00<br>05:23.40<br>−0.80	08:27.60<br>08:27.35<br>−0.05	14:28.20<br>14:28.92<br>0.08	30:06.20<br>30:05.59<br>−0.03	0.40	1:06:16.68	2:29:01.80<br>2:18:12.43<br>−7.26
1926	05:55.40	366.00	0.48	5.63	0.137	0.058	02:26.80<br>02:26.11<br>−0.47	03:51.00<br>03:52.35<br>0.58	04:10.40<br>04:11.93<br>0.61	05:26.00<br>05:23.72<br>−0.70	08:20.40<br>08:21.82<br>0.28	14:28.20<br>14:24.08<br>−0.47	30:06.20<br>30:09.77<br>0.20	0.47	1:07:03.48	2:29:01.80<br>2:21:11.49<br>−5.26
1927	06:09.69	365.17	0.49	5.61	0.138	0.058	02:25.80<br>02:25.55<br>−0.17	03:51.00<br>03:51.48<br>0.21	04:10.40<br>04:10.99<br>0.24	05:23.40<br>05:22.53<br>−0.27	08:20.40<br>08:21.82<br>0.28	14:28.20<br>14:24.08<br>−0.47	30:06.20<br>30:09.77<br>0.20	0.26	1:07:03.48	2:29:01.80<br>2:21:11.49<br>−5.26
1928	06:09.69	365.17	0.49	5.61	0.138	0.058	02:25.80<br>02:25.55<br>−0.17	03:51.00<br>03:51.48<br>0.21	04:10.40<br>04:10.99<br>0.24	05:23.40<br>05:22.53<br>−0.27	08:20.40<br>08:21.82<br>0.28	14:28.20<br>14:24.08<br>−0.47	30:06.20<br>30:09.77<br>0.20	0.26	1:07:03.48	2:29:01.80<br>2:21:11.49<br>−5.26
1929	06:09.69	365.17	0.49	5.61	0.138	0.058	02:25.80<br>02:25.55<br>−0.17	03:51.00<br>03:51.48<br>0.21	04:10.40<br>04:10.99<br>0.24	05:23.40<br>05:22.53<br>−0.27	08:20.40<br>08:21.82<br>0.28	14:28.20<br>14:24.08<br>−0.47	30:06.20<br>30:09.77<br>0.20	0.26	1:07:03.48	2:29:01.80<br>2:21:11.49<br>−5.26
1930	05:55.06	366.02	0.53	5.63	0.158	0.058	02:23.60<br>02:23.42<br>−0.13	03:49.20<br>03:50.15<br>0.41	04:10.40<br>04:09.98<br>−0.17	05:23.40<br>05:23.03<br>−0.11	08:20.40<br>08:21.82<br>0.28	14:28.20<br>14:24.08<br>−0.47	30:06.20<br>30:09.77<br>0.20	0.25	1:07:03.48	2:29:01.80<br>2:21:11.49<br>−5.26
1931	06:13.33	364.96	0.52	5.60	0.152	0.058	02:23.60<br>02:23.49<br>−0.08	03:49.20<br>03:49.59<br>0.17	04:09.20<br>04:09.23<br>0.01	05:21.80<br>05:21.48<br>−0.10	08:20.40<br>08:21.82<br>0.28	14:28.20<br>14:24.08<br>−0.47	30:06.20<br>30:09.77<br>0.20	0.19	1:07:03.48	2:29:01.80<br>2:21:11.49<br>−5.26
1932	05:41.92	369.85	0.51	5.20	0.150	0.061	02:23.60<br>02:23.49<br>−0.08	03:49.20<br>03:49.59<br>0.17	04:09.20<br>04:09.23<br>0.01	05:21.80<br>05:21.48<br>−0.10	08:18.80<br>08:18.05<br>−0.15	14:17.00<br>14:19.17<br>0.25	30:06.20<br>30:04.37<br>−0.10	0.12	1:07:04.64	2:29:01.80<br>2:21:43.02<br>−4.91
1933	05:50.22	369.31	0.51	5.19	0.149	0.061	02:23.60<br>02:23.33<br>−0.19	03:49.00<br>03:49.17<br>0.07	04:07.60<br>04:08.74<br>0.46	05:21.80<br>05:20.71<br>−0.34	08:18.80<br>08:18.05<br>−0.15	14:17.00<br>14:19.17<br>0.25	30:06.20<br>30:04.37<br>−0.10	0.22	1:07:04.64	2:29:01.80<br>2:21:43.02<br>−4.91
1934	05:51.82	369.48	0.51	5.12	0.149	0.061	02:23.60<br>02:23.23<br>−0.26	03:48.80<br>03:48.94<br>0.06	04:06.80<br>04:08.47<br>0.68	05:21.80<br>05:20.30<br>−0.47	08:18.40<br>08:17.74<br>−0.13	14:17.00<br>14:18.90<br>0.22	30:06.20<br>30:04.60<br>−0.09	0.27	1:07:07.31	2:29:01.80<br>2:21:53.44<br>−4.79

Year	t_c	v_m [m/min]	E_s	E_l	γ_{an}	γ_{ae}	1000	1500	1609.34	2000	3000	5000	10 000	av.err [%]	Halfmarathon	Marathon
1935	05:51.82	369.48	0.51	5.12	0.149	0.061	02:23.60<br>02:23.23<br>−0.26	03:48.80<br>03:48.94<br>0.06	04:06.80<br>04:08.47<br>0.68	05:21.80<br>05:20.30<br>−0.47	08:18.40<br>08:17.74<br>−0.13	14:17.00<br>14:18.90<br>0.22	30:06.20<br>30:04.60<br>−0.09	0.27	1:07:07.31	2:26:42.00<br>2:21:53.44<br>−3.28
1936	05:39.14	372.85	0.50	4.62	0.143	0.065	02:23.60<br>02:23.22<br>−0.27	03:47.80<br>03:48.44<br>0.28	04:06.80<br>04:07.83<br>0.42	05:20.40<br>05:19.05<br>−0.42	08:14.80<br>08:14.99<br>0.04	14:17.00<br>14:16.44<br>−0.07	30:06.20<br>30:06.68<br>0.03	0.22	1:07:31.67	2:26:42.00<br>2:23:29.31<br>−2.19
1937	06:20.54	370.01	0.47	4.59	0.133	0.066	02:23.60<br>02:23.56<br>−0.03	03:47.80<br>03:47.70<br>−0.05	04:06.40<br>04:06.76<br>0.15	05:16.80<br>05:16.57<br>−0.07	08:14.80<br>08:15.02<br>0.04	14:17.00<br>14:16.37<br>−0.07	30:05.60<br>30:06.14<br>0.03	0.06	1:07:29.43	2:26:42.00<br>2:23:22.20<br>−2.27
1938	06:24.15	369.54	0.47	4.73	0.133	0.064	02:23.60<br>02:23.56<br>−0.03	03:47.80<br>03:47.70<br>−0.05	04:06.40<br>04:06.76<br>0.15	05:16.80<br>05:16.57<br>−0.07	08:14.80<br>08:15.17<br>0.08	14:17.00<br>14:15.92<br>−0.13	30:02.00<br>30:02.92<br>0.05	0.08	1:07:16.03	2:26:42.00<br>2:22:39.77<br>−2.75
1939	06:13.19	370.97	0.47	5.08	0.133	0.061	02:23.60<br>02:23.56<br>−0.03	03:47.80<br>03:47.70<br>−0.05	04:06.40<br>04:06.76<br>0.15	05:16.80<br>05:16.57<br>−0.07	08:14.80<br>08:13.71<br>−0.22	14:08.80<br>14:11.94<br>0.37	29:52.60<br>29:49.97<br>−0.15	0.15	1:06:34.62	2:26:42.00<br>2:20:44.28<br>−4.06
1940	05:16.80	379.03	0.46	4.39	0.130	0.068	02:23.60<br>02:23.56<br>−0.03	03:47.80<br>03:47.69<br>−0.05	04:06.40<br>04:06.76<br>0.15	05:16.80<br>05:16.57<br>−0.07	08:09.00<br>08:09.29<br>0.06	14:08.80<br>14:07.98<br>−0.10	29:52.60<br>29:53.30<br>0.04	0.07	1:07:14.02	2:26:42.00<br>2:23:19.95<br>−2.30
1941	04:55.94	380.62	0.52	4.44	0.154	0.067	02:21.50<br>02:21.54<br>0.03	03:47.60<br>03:47.19<br>−0.18	04:06.40<br>04:06.78<br>0.15	05:16.80<br>05:16.71<br>−0.03	08:09.00<br>08:09.42<br>0.09	14:08.80<br>14:08.08<br>−0.08	29:52.60<br>29:53.07<br>0.03	0.08	1:07:12.30	2:26:42.00<br>2:23:13.52<br>−2.37
1942	04:21.74	390.90	0.48	3.86	0.138	0.074	02:21.50<br>02:21.52<br>0.01	03:45.80<br>03:45.63<br>−0.08	04:04.60<br>04:04.76<br>0.07	05:11.80<br>05:10.95<br>−0.27	08:01.20<br>08:02.30<br>0.23	13:58.20<br>13:59.98<br>0.21	29:52.60<br>29:49.67<br>−0.16	0.15	1:07:43.44	2:26:42.00<br>2:25:50.60<br>−0.58
1943	04:56.55	387.29	0.46	3.81	0.128	0.075	02:21.50<br>02:21.56<br>0.04	03:45.00<br>03:44.40<br>−0.27	04:02.60<br>04:03.16<br>0.23	05:11.80<br>05:10.95<br>−0.27	08:01.20<br>08:02.30<br>0.23	13:58.20<br>13:59.98<br>0.21	29:52.60<br>29:49.67<br>−0.16	0.20	1:07:43.44	2:26:42.00<br>2:25:50.60<br>−0.58
1944	05:17.35	385.21	0.46	4.05	0.128	0.072	02:21.50<br>02:21.17<br>−0.23	03:43.00<br>03:43.66<br>0.29	04:01.60<br>04:02.33<br>0.30	05:11.80<br>05:10.68<br>−0.36	08:01.20<br>08:01.66<br>0.10	13:58.20<br>13:56.86<br>−0.16	29:35.40<br>29:36.54<br>0.06	0.22	1:06:55.26	2:26:42.00<br>2:23:23.76<br>−2.25
1945	05:18.74	385.09	0.46	4.04	0.127	0.072	02:21.50<br>02:21.15<br>−0.24	03:43.00<br>03:43.61<br>0.27	04:01.40<br>04:02.28<br>0.36	05:11.80<br>05:10.59<br>−0.39	08:01.20<br>08:01.66<br>0.10	13:58.20<br>13:56.86<br>−0.16	29:35.40<br>29:36.54<br>0.06	0.23	1:06:55.26	2:26:42.00<br>2:23:23.76<br>−2.25
1946	05:17.84	385.16	0.46	4.04	0.128	0.072	02:21.40<br>02:21.07<br>−0.23	03:43.00<br>03:43.57<br>0.26	04:01.40<br>04:02.26<br>0.35	05:11.80<br>05:10.64<br>−0.37	08:01.20<br>08:01.66<br>0.10	13:58.20<br>13:56.86<br>−0.16	29:35.40<br>29:36.54<br>0.06	0.22	1:06:55.26	2:26:42.00<br>2:23:23.76<br>−2.25
1947	05:17.84	385.16	0.46	4.04	0.128	0.072	02:21.40<br>02:21.07<br>−0.23	03:43.00<br>03:43.57<br>0.26	04:01.40<br>04:02.26<br>0.35	05:11.80<br>05:10.64<br>−0.37	08:01.20<br>08:01.66<br>0.10	13:58.20<br>13:56.86<br>−0.16	29:35.40<br>29:36.54<br>0.06	0.22	1:06:55.26	2:25:39.00<br>2:23:23.76<br>−1.55
1948	06:36.54	379.07	0.42	3.96	0.115	0.073	02:21.40<br>02:21.57<br>0.12	03:43.00<br>03:42.69<br>−0.14	04:01.40<br>04:00.97<br>−0.18	05:07.00<br>05:07.61<br>0.20	08:01.20<br>08:01.66<br>0.10	13:58.20<br>13:56.86<br>−0.16	29:35.40<br>29:36.54<br>0.06	0.14	1:06:55.26	2:25:39.00<br>2:23:23.76<br>−1.55
1949	06:19.63	380.96	0.42	4.16	0.114	0.070	02:21.40<br>02:21.57<br>0.12	03:43.00<br>03:42.69<br>−0.14	04:01.40<br>04:00.97<br>−0.18	05:07.00<br>05:07.61<br>0.20	07:58.80<br>08:00.43<br>0.34	13:58.20<br>13:53.47<br>−0.56	29:21.20<br>29:25.32<br>0.23	0.25	1:06:18.63	2:25:39.00<br>2:21:39.22<br>−2.74
1950	06:44.18	378.24	0.42	4.84	0.115	0.063	02:21.40<br>02:21.57<br>0.12	03:43.00<br>03:42.69<br>−0.14	04:01.40<br>04:00.97<br>−0.18	05:07.00<br>05:07.61<br>0.20	07:58.80<br>08:01.22<br>0.50	13:58.20<br>13:51.16<br>−0.84	29:02.60<br>29:08.79<br>0.36	0.33	1:05:09.44	2:25:39.00<br>2:17:59.19<br>−5.26
1951	06:44.18	378.24	0.42	4.84	0.115	0.063	02:21.40<br>02:21.57<br>0.12	03:43.00<br>03:42.69<br>−0.14	04:01.40<br>04:00.97<br>−0.18	05:07.00<br>05:07.61<br>0.20	07:58.80<br>08:01.22<br>0.50	13:58.20<br>13:51.16<br>−0.84	29:02.60<br>29:08.79<br>0.36	0.33	1:05:09.44	2:25:39.00<br>2:17:59.19<br>−5.26

Year	t_c	v_m [m/min]	E_s	E_l	γ_{an}	γ_{ae}	1000	1500	1609.34	2000	3000	5000	10 000	av.err [%]	Halfmarathon	Marathon
1952	06:37.95	378.61	0.42	4.85	0.117	0.063	02:21.20<br>02:21.40<br>0.14	03:43.00<br>03:42.62<br>−0.17	04:01.40<br>04:00.93<br>−0.20	05:07.00<br>05:07.71<br>0.23	07:58.80<br>08:01.22<br>0.50	13:58.20<br>13:51.16<br>−0.84	29:02.60<br>29:08.79<br>0.36	0.35	1:05:09.44	2:20:42.20<br>2:17:59.19<br>−1.93
1953	06:18.77	379.73	0.45	4.92	0.124	0.063	02:20.40<br>02:20.71<br>0.22	03:43.00<br>03:42.31<br>−0.31	04:01.40<br>04:00.75<br>−0.27	05:07.00<br>05:08.12<br>0.36	07:58.80<br>08:01.26<br>0.51	13:58.20<br>13:51.04<br>−0.85	29:01.60<br>29:07.91<br>0.36	0.41	1:05:05.77	2:18:34.80<br>2:17:47.62<br>−0.57
1954	06:25.61	380.04	0.46	5.21	0.130	0.061	02:19.50<br>02:19.50<br>0.00	03:41.80<br>03:40.87<br>−0.42	03:58.00<br>03:59.29<br>0.54	05:07.00<br>05:06.65<br>−0.11	07:58.80<br>08:00.00<br>0.25	13:51.20<br>13:47.70<br>−0.42	28:54.20<br>28:57.23<br>0.17	0.27	1:04:32.02	2:17:39.40<br>2:16:14.25<br>−1.03
1955	06:34.83	383.49	0.43	4.58	0.117	0.066	02:19.00<br>02:19.41<br>0.29	03:40.80<br>03:39.55<br>−0.57	03:58.00<br>03:57.62<br>−0.16	05:02.20<br>05:03.54<br>0.44	07:55.60<br>07:55.15<br>−0.09	13:40.60<br>13:41.90<br>0.16	28:54.20<br>28:53.10<br>−0.06	0.25	1:04:44.78	2:17:39.40<br>2:17:30.38<br>−0.11
1956	06:16.82	385.68	0.42	5.28	0.117	0.060	02:19.00<br>02:19.38<br>0.27	03:40.60<br>03:39.50<br>−0.50	03:58.00<br>03:57.56<br>−0.18	05:02.20<br>05:03.46<br>0.42	07:52.80<br>07:53.18<br>0.08	13:36.80<br>13:35.70<br>−0.13	28:30.40<br>28:31.34<br>0.05	0.23	1:03:32.42	2:17:39.40<br>2:14:04.22<br>−2.61
1957	06:38.33	384.72	0.42	5.22	0.116	0.060	02:19.00<br>02:18.94<br>−0.04	03:38.10<br>03:38.69<br>0.27	03:57.20<br>03:56.66<br>−0.23	05:02.20<br>05:02.21<br>0.00	07:52.80<br>07:52.77<br>−0.01	13:35.00<br>13:35.09<br>0.01	28:30.40<br>28:30.33<br>−0.00	0.08	1:03:30.91	2:17:39.40<br>2:14:02.69<br>−2.62
1958	06:55.35	383.75	0.44	5.20	0.123	0.061	02:18.10<br>02:17.63<br>−0.34	03:36.00<br>03:37.19<br>0.55	03:54.50<br>03:55.15<br>0.28	05:02.20<br>05:00.75<br>−0.48	07:52.80<br>07:52.77<br>−0.01	13:35.00<br>13:35.09<br>0.01	28:30.40<br>28:30.33<br>−0.00	0.24	1:03:30.91	2:15:17.00<br>2:14:02.69<br>−0.92
1959	06:46.65	384.24	0.45	5.21	0.126	0.061	02:17.80<br>02:17.38<br>−0.30	03:36.00<br>03:37.07<br>0.50	03:54.50<br>03:55.08<br>0.25	05:02.20<br>05:00.89<br>−0.43	07:52.80<br>07:52.77<br>−0.01	13:35.00<br>13:35.09<br>0.01	28:30.40<br>28:30.33<br>−0.00	0.21	1:03:30.91	2:15:17.00<br>2:14:02.69<br>−0.92
1960	06:33.21	384.30	0.48	6.02	0.137	0.056	02:16.70<br>02:16.39<br>−0.22	03:35.60<br>03:36.54<br>0.44	03:54.50<br>03:54.72<br>0.09	05:02.20<br>05:01.30<br>−0.30	07:52.80<br>07:53.28<br>0.10	13:35.00<br>13:33.62<br>−0.17	28:18.80<br>28:19.98<br>0.07	0.20	1:02:48.57	2:15:16.20<br>2:11:51.15<br>−2.53
1961	06:33.21	384.30	0.48	6.02	0.137	0.056	02:16.70<br>02:16.39<br>−0.22	03:35.60<br>03:36.54<br>0.44	03:54.50<br>03:54.72<br>0.09	05:02.20<br>05:01.30<br>−0.30	07:52.80<br>07:53.28<br>0.10	13:35.00<br>13:33.62<br>−0.17	28:18.80<br>28:19.98<br>0.07	0.20	1:02:48.57	2:15:16.20<br>2:11:51.15<br>−2.53
1962	06:10.19	388.12	0.47	5.30	0.133	0.060	02:16.70<br>02:16.45<br>−0.18	03:35.60<br>03:36.41<br>0.38	03:54.40<br>03:54.53<br>0.05	05:01.60<br>05:00.87<br>−0.24	07:49.20<br>07:50.54<br>0.29	13:35.00<br>13:31.11<br>−0.48	28:18.20<br>28:21.57<br>0.20	0.26	1:03:10.31	2:15:16.20<br>2:13:16.80<br>−1.47
1963	06:12.58	387.79	0.47	5.45	0.133	0.059	02:16.70<br>02:16.45<br>−0.18	03:35.60<br>03:36.41<br>0.38	03:54.40<br>03:54.53<br>0.05	05:01.60<br>05:00.87<br>−0.24	07:49.20<br>07:50.65<br>0.31	13:35.00<br>13:30.78<br>−0.52	28:15.60<br>28:19.26<br>0.22	0.27	1:03:00.82	2:14:28.00<br>2:12:47.20<br>−1.25
1964	06:13.50	387.74	0.47	5.45	0.134	0.059	02:16.60<br>02:16.34<br>−0.19	03:35.60<br>03:36.29<br>0.32	03:54.04<br>03:54.41<br>0.16	05:01.60<br>05:00.76<br>−0.28	07:49.20<br>07:50.65<br>0.31	13:35.00<br>13:30.78<br>−0.52	28:15.60<br>28:19.26<br>0.22	0.28	1:03:00.82	2:12:11.20<br>2:12:47.20<br>0.45
1965	05:02.11	399.04	0.47	5.71	0.132	0.057	02:16.20<br>02:16.01<br>−0.14	03:35.60<br>03:35.95<br>0.16	03:53.60<br>03:54.09<br>0.21	05:01.20<br>05:00.51<br>−0.23	07:39.60<br>07:42.38<br>0.61	13:24.20<br>13:16.09<br>−1.01	27:39.89<br>27:47.08<br>0.43	0.40	1:01:45.49	2:12:00.00<br>2:10:00.30<br>−1.51
1966	05:54.01	396.85	0.42	5.51	0.114	0.059	02:16.20<br>02:16.33<br>0.10	03:35.60<br>03:34.51<br>−0.50	03:51.30<br>03:52.13<br>0.36	04:56.20<br>04:56.36<br>0.06	07:39.60<br>07:40.68<br>0.23	13:16.60<br>13:13.47<br>−0.39	27:39.89<br>27:42.59<br>0.16	0.26	1:01:38.17	2:12:00.00<br>2:09:50.74<br>−1.63
1967	06:13.82	395.59	0.42	5.48	0.114	0.059	02:16.20<br>02:15.94<br>−0.19	03:33.10<br>03:33.84<br>0.35	03:51.10<br>03:51.39<br>0.13	04:56.20<br>04:55.39<br>−0.28	07:39.60<br>07:40.68<br>0.23	13:16.60<br>13:13.47<br>−0.39	27:39.89<br>27:42.59<br>0.16	0.25	1:01:38.17	2:09:36.40<br>2:09:50.74<br>0.18
1968	06:13.82	395.59	0.42	5.48	0.114	0.059	02:16.20<br>02:15.94<br>−0.19	03:33.10<br>03:33.84<br>0.35	03:51.10<br>03:51.39<br>0.13	04:56.20<br>04:55.39<br>−0.28	07:39.60<br>07:40.68<br>0.23	13:16.60<br>13:13.47<br>−0.39	27:39.89<br>27:42.59<br>0.16	0.25	1:01:38.17	2:09:36.40<br>2:09:50.74<br>0.18

Year	t_c	v_m [m/min]	E_s	E_l	γ_{an}	γ_{ae}	1000	1500	1609.34	2000	3000	5000	10 000	av.err [%]	Halfmarathon	Marathon
1969	06:13.82	395.59	0.42	5.48	0.114	0.059	02:16.20<br>02:15.94<br>−0.19	03:33.10<br>03:33.84<br>0.35	03:51.10<br>03:51.39<br>0.13	04:56.20<br>04:55.39<br>−0.28	07:39.60<br>07:40.68<br>0.23	13:16.60<br>13:13.47<br>−0.39	27:39.89<br>27:42.59<br>0.16	0.25	1:01:38.17	2:08:33.60<br>2:09:50.74<br>1.00
1970	06:13.82	395.59	0.42	5.48	0.114	0.059	02:16.20<br>02:15.94<br>−0.19	03:33.10<br>03:33.84<br>0.35	03:51.10<br>03:51.39<br>0.13	04:56.20<br>04:55.39<br>−0.28	07:39.60<br>07:40.68<br>0.23	13:16.60<br>13:13.47<br>−0.39	27:39.89<br>27:42.59<br>0.16	0.25	1:01:38.17	2:08:33.60<br>2:09:50.74<br>1.00
1971	06:13.82	395.59	0.42	5.48	0.114	0.059	02:16.20<br>02:15.94<br>−0.19	03:33.10<br>03:33.84<br>0.35	03:51.10<br>03:51.39<br>0.13	04:56.20<br>04:55.39<br>−0.28	07:39.60<br>07:40.68<br>0.23	13:16.60<br>13:13.47<br>−0.39	27:39.89<br>27:42.59<br>0.16	0.25	1:01:38.17	2:08:33.60<br>2:09:50.74<br>1.00
1972	05:39.03	400.01	0.41	5.18	0.113	0.061	02:16.20<br>02:15.94<br>−0.19	03:33.10<br>03:33.84<br>0.35	03:51.10<br>03:51.39<br>0.13	04:56.20<br>04:55.39<br>−0.28	07:37.60<br>07:38.40<br>0.17	13:13.00<br>13:10.69<br>−0.29	27:38.35<br>27:40.34<br>0.12	0.22	1:01:42.77	2:08:33.60<br>2:10:21.64<br>1.40
1973	05:45.30	398.94	0.42	5.65	0.115	0.058	02:16.00<br>02:15.77<br>−0.17	03:33.10<br>03:33.76<br>0.31	03:51.10<br>03:51.35<br>0.11	04:56.20<br>04:55.48<br>−0.24	07:37.60<br>07:38.72<br>0.25	13:13.00<br>13:09.74<br>−0.41	27:30.80<br>27:33.62<br>0.17	0.24	1:01:15.16	2:08:33.60<br>2:08:55.55<br>0.28
1974	05:01.83	404.11	0.48	5.23	0.134	0.060	02:13.90<br>02:13.84<br>−0.04	03:32.16<br>03:32.71<br>0.26	03:51.10<br>03:50.60<br>−0.21	04:56.20<br>04:56.20<br>0.00	07:35.20<br>07:36.88<br>0.37	13:13.00<br>13:08.12<br>−0.62	27:30.80<br>27:35.06<br>0.26	0.25	1:01:31.34	2:08:33.60<br>2:09:58.26<br>1.10
1975	05:11.26	403.35	0.47	5.21	0.133	0.061	02:13.90<br>02:13.71<br>−0.14	03:32.16<br>03:32.31<br>0.07	03:49.40<br>03:50.14<br>0.32	04:56.20<br>04:55.45<br>−0.25	07:35.20<br>07:36.88<br>0.37	13:13.00<br>13:08.12<br>−0.62	27:30.80<br>27:35.06<br>0.26	0.29	1:01:31.34	2:08:33.60<br>2:09:58.26<br>1.10
1976	06:09.69	399.15	0.43	5.12	0.119	0.061	02:13.90<br>02:14.19<br>0.21	03:32.16<br>03:31.46<br>−0.33	03:49.40<br>03:48.90<br>−0.22	04:51.52<br>04:52.51<br>0.34	07:35.20<br>07:36.88<br>0.37	13:13.00<br>13:08.12<br>−0.62	27:30.80<br>27:35.06<br>0.26	0.33	1:01:31.34	2:08:33.60<br>2:09:58.26<br>1.10
1977	06:09.67	399.15	0.43	5.14	0.119	0.061	02:13.90<br>02:14.19<br>0.21	03:32.16<br>03:31.46<br>−0.33	03:49.40<br>03:48.90<br>−0.22	04:51.52<br>04:52.51<br>0.34	07:35.20<br>07:36.86<br>0.37	13:12.87<br>13:08.03<br>−0.61	27:30.47<br>27:34.69<br>0.26	0.33	1:01:30.01	2:08:33.60<br>2:09:54.33<br>1.05
1978	05:28.75	404.71	0.43	5.03	0.117	0.062	02:13.90<br>02:14.19<br>0.21	03:32.16<br>03:31.46<br>−0.33	03:49.40<br>03:48.90<br>−0.22	04:51.52<br>04:52.51<br>0.34	07:32.10<br>07:33.82<br>0.38	13:08.40<br>13:03.39<br>−0.64	27:22.47<br>27:26.84<br>0.27	0.34	1:01:17.64	2:08:33.60<br>2:09:39.60<br>0.86
1979	05:33.71	404.33	0.42	5.02	0.117	0.062	02:13.90<br>02:14.13<br>0.17	03:32.03<br>03:31.33<br>−0.33	03:48.95<br>03:48.74<br>−0.09	04:51.52<br>04:52.26<br>0.25	07:32.10<br>07:33.82<br>0.38	13:08.40<br>13:03.39<br>−0.64	27:22.47<br>27:26.84<br>0.27	0.31	1:01:17.64	2:08:33.60<br>2:09:39.60<br>0.86
1980	05:31.06	404.53	0.44	5.02	0.121	0.062	02:13.40<br>02:13.59<br>0.15	03:31.36<br>03:30.93<br>−0.20	03:48.80<br>03:48.40<br>−0.18	04:51.52<br>04:52.21<br>0.24	07:32.10<br>07:33.82<br>0.38	13:08.40<br>13:03.39<br>−0.64	27:22.47<br>27:26.84<br>0.27	0.29	1:01:17.64	2:08:33.60<br>2:09:39.60<br>0.86
1981	05:20.04	405.83	0.47	5.02	0.132	0.062	02:12.18<br>02:12.44<br>0.20	03:31.36<br>03:30.11<br>−0.59	03:47.33<br>03:47.72<br>0.17	04:51.52<br>04:52.19<br>0.23	07:32.10<br>07:33.33<br>0.27	13:06.20<br>13:02.63<br>−0.45	27:22.47<br>27:25.56<br>0.19	0.30	1:01:15.61	2:08:18.00<br>2:09:37.16<br>1.03
1982	05:06.79	408.09	0.47	4.96	0.131	0.062	02:12.18<br>02:12.44<br>0.20	03:31.36<br>03:30.11<br>−0.59	03:47.33<br>03:47.72<br>0.17	04:51.52<br>04:52.19<br>0.23	07:32.10<br>07:32.02<br>−0.02	13:00.41<br>13:00.65<br>0.03	27:22.47<br>27:22.27<br>−0.01	0.18	1:01:10.63	2:08:18.00<br>2:09:31.94<br>0.96
1983	05:09.75	407.84	0.47	4.96	0.131	0.062	02:12.18<br>02:12.36<br>0.13	03:30.77<br>03:29.97<br>−0.38	03:47.33<br>03:47.56<br>0.10	04:51.52<br>04:51.97<br>0.15	07:32.10<br>07:32.02<br>−0.02	13:00.41<br>13:00.65<br>0.03	27:22.47<br>27:22.27<br>−0.01	0.12	1:01:10.63	2:08:18.00<br>2:09:31.94<br>0.96
1984	05:19.08	406.26	0.47	5.44	0.132	0.059	02:12.18<br>02:12.36<br>0.13	03:30.77<br>03:29.97<br>−0.38	03:47.33<br>03:47.56<br>0.10	04:51.52<br>04:51.97<br>0.15	07:32.10<br>07:32.39<br>0.06	13:00.41<br>12:59.56<br>−0.11	27:13.81<br>27:14.54<br>0.04	0.14	1:00:38.71	2:08:05.00<br>2:07:51.95<br>−0.17
1985	05:33.38	405.21	0.47	5.42	0.131	0.059	02:12.18<br>02:12.09<br>−0.07	03:29.46<br>03:29.38<br>−0.04	03:46.32<br>03:46.89<br>0.25	04:51.39<br>04:50.97<br>−0.14	07:32.10<br>07:32.39<br>0.06	13:00.40<br>12:59.56<br>−0.11	27:13.81<br>27:14.53<br>0.04	0.10	1:00:55.00<br>1:00:38.71<br>−0.45	2:07:12.00<br>2:07:51.94<br>0.52

Year	t_c	v_m [m/min]	E_s	E_l	γ_{an}	γ_{ae}	1000	1500	1609.34	2000	3000	5000	10 000	av.err [%]	Halfmarathon	Marathon
1986	05:33.38	405.21	0.47	5.42	0.131	0.059	02:12.18<br>02:12.09<br>−0.07	03:29.46<br>03:29.38<br>−0.04	03:46.32<br>03:46.89<br>0.25	04:51.39<br>04:50.97<br>−0.14	07:32.10<br>07:32.39<br>0.06	13:00.40<br>12:59.56<br>−0.11	27:13.81<br>27:14.53<br>0.04	0.10	1:00:43.00<br>1:00:38.71<br>−0.12	2:07:12.00<br>2:07:51.94<br>0.52
1987	05:35.22	405.49	0.46	5.38	0.129	0.059	02:12.18<br>02:12.15<br>−0.02	03:29.46<br>03:29.28<br>−0.09	03:46.32<br>03:46.73<br>0.18	04:50.81<br>04:50.60<br>−0.07	07:32.10<br>07:31.93<br>−0.04	12:58.39<br>12:58.87<br>0.06	27:13.81<br>27:13.40<br>−0.02	0.07	1:00:10.00<br>1:00:37.03<br>0.75	2:07:12.00<br>2:07:50.26<br>0.50
1988	05:35.22	405.49	0.46	5.38	0.129	0.059	02:12.18<br>02:12.15<br>−0.02	03:29.46<br>03:29.28<br>−0.09	03:46.32<br>03:46.73<br>0.18	04:50.81<br>04:50.60<br>−0.07	07:32.10<br>07:31.93<br>−0.04	12:58.39<br>12:58.87<br>0.06	27:13.81<br>27:13.40<br>−0.02	0.07	1:00:10.00<br>1:00:37.03<br>0.75	2:06:50.00<br>2:07:50.26<br>0.79
1989	05:15.55	408.64	0.46	5.24	0.128	0.060	02:12.18<br>02:12.15<br>−0.02	03:29.46<br>03:29.28<br>−0.09	03:46.32<br>03:46.73<br>0.18	04:50.81<br>04:50.60<br>−0.07	07:29.45<br>07:30.15<br>0.16	12:58.39<br>12:56.37<br>−0.26	27:08.23<br>27:09.97<br>0.11	0.13	1:00:10.00<br>1:00:34.25<br>0.67	2:06:50.00<br>2:07:55.15<br>0.86
1990	05:15.55	408.64	0.46	5.24	0.128	0.060	02:12.18<br>02:12.15<br>−0.02	03:29.46<br>03:29.28<br>−0.09	03:46.32<br>03:46.73<br>0.18	04:50.81<br>04:50.60<br>−0.07	07:29.45<br>07:30.15<br>0.16	12:58.39<br>12:56.37<br>−0.26	27:08.23<br>27:09.97<br>0.11	0.13	1:00:10.00<br>1:00:34.25<br>0.67	2:06:50.00<br>2:07:55.15<br>0.86
1991	05:15.55	408.64	0.46	5.24	0.128	0.060	02:12.18<br>02:12.15<br>−0.02	03:29.46<br>03:29.28<br>−0.09	03:46.32<br>03:46.73<br>0.18	04:50.81<br>04:50.60<br>−0.07	07:29.45<br>07:30.15<br>0.16	12:58.39<br>12:56.37<br>−0.26	27:08.23<br>27:09.97<br>0.11	0.13	1:00:10.00<br>1:00:34.25<br>0.67	2:06:50.00<br>2:07:55.15<br>0.86
1992	05:13.75	409.20	0.46	5.15	0.128	0.061	02:12.18<br>02:12.06<br>−0.09	03:28.86<br>03:29.13<br>0.13	03:46.32<br>03:46.57<br>0.11	04:50.81<br>04:50.39<br>−0.14	07:28.96<br>07:29.77<br>0.18	12:58.39<br>12:56.04<br>−0.30	27:08.23<br>27:10.26<br>0.12	0.15	1:00:10.00<br>1:00:37.55<br>0.76	2:06:50.00<br>2:08:08.04<br>1.03
1993	05:37.45	406.53	0.45	5.75	0.127	0.057	02:12.18<br>02:11.90<br>−0.22	03:28.86<br>03:28.68<br>−0.09	03:44.39<br>03:46.05<br>0.74	04:50.81<br>04:49.57<br>−0.43	07:28.96<br>07:30.20<br>0.28	12:58.39<br>12:54.79<br>−0.46	26:58.38<br>27:01.50<br>0.19	0.34	59:47.00<br>1:00:01.55<br>0.41	2:06:50.00<br>2:06:15.78<br>−0.45
1994	04:58.11	412.91	0.45	5.13	0.125	0.061	02:12.18<br>02:11.90<br>−0.22	03:28.86<br>03:28.68<br>−0.09	03:44.39<br>03:46.05<br>0.74	04:50.81<br>04:49.57<br>−0.43	07:25.11<br>07:27.01<br>0.43	12:56.96<br>12:51.43<br>−0.71	26:56.23<br>27:01.07<br>0.30	0.42	59:47.00<br>1:00:18.38<br>0.87	2:06:50.00<br>2:07:30.47<br>0.53
1995	05:22.76	412.34	0.42	5.71	0.115	0.057	02:12.18<br>02:11.98<br>−0.15	03:27.37<br>03:27.77<br>0.19	03:44.39<br>03:44.86<br>0.21	04:47.88<br>04:47.18<br>−0.24	07:25.11<br>07:24.72<br>−0.09	12:44.39<br>12:45.52<br>0.15	26:43.53<br>26:42.58<br>−0.06	0.16	59:47.00<br>59:20.82<br>−0.73	2:06:50.00<br>2:04:52.91<br>−1.54
1996	04:47.88	417.98	0.41	5.32	0.112	0.060	02:12.18<br>02:12.01<br>−0.13	03:27.37<br>03:27.72<br>0.17	03:44.39<br>03:44.78<br>0.17	04:47.88<br>04:47.00<br>−0.31	07:20.67<br>07:21.98<br>0.30	12:44.39<br>12:42.13<br>−0.30	26:38.08<br>26:39.59<br>0.09	0.21	59:47.00<br>59:25.19<br>−0.61	2:06:50.00<br>2:05:26.40<br>−1.10
1997	04:47.88	417.93	0.41	5.93	0.112	0.056	02:12.18<br>02:12.00<br>−0.14	03:27.37<br>03:27.73<br>0.17	03:44.39<br>03:44.80<br>0.18	04:47.88<br>04:47.04<br>−0.29	07:20.67<br>07:21.28<br>0.14	12:39.74<br>12:39.18<br>−0.07	26:27.85<br>26:27.98<br>0.01	0.14	59:47.00<br>58:44.87<br>−1.73	2:06:50.00<br>2:03:29.61<br>−2.63
1998	04:53.78	417.38	0.41	6.20	0.114	0.055	02:12.18<br>02:11.76<br>−0.32	03:26.00<br>03:27.43<br>0.70	03:44.39<br>03:44.49<br>0.04	04:47.88<br>04:46.71<br>−0.41	07:20.67<br>07:21.08<br>0.09	12:39.36<br>12:38.16<br>−0.16	26:22.75<br>26:23.78<br>0.06	0.25	59:17.00<br>58:30.05<br>−1.32	2:06:05.00<br>2:02:46.52<br>−2.62
1999	05:45.46	413.68	0.38	6.10	0.104	0.055	02:11.96<br>02:11.80<br>−0.12	03:26.00<br>03:26.48<br>0.23	03:43.13<br>03:43.26<br>0.06	04:44.79<br>04:44.31<br>−0.17	07:20.67<br>07:21.08<br>0.09	12:39.36<br>12:38.16<br>−0.16	26:22.75<br>26:23.78<br>0.06	0.13	59:17.00<br>58:30.05<br>−1.32	2:05:42.00<br>2:02:46.52<br>−2.33
2000	05:45.46	413.68	0.38	6.10	0.104	0.055	02:11.96<br>02:11.80<br>−0.12	03:26.00<br>03:26.48<br>0.23	03:43.13<br>03:43.26<br>0.06	04:44.79<br>04:44.31<br>−0.17	07:20.67<br>07:21.08<br>0.09	12:39.36<br>12:38.16<br>−0.16	26:22.75<br>26:23.78<br>0.06	0.13	59:06.00<br>58:30.05<br>−1.01	2:05:42.00<br>2:02:46.52<br>−2.33
2001	05:45.46	413.68	0.38	6.10	0.104	0.055	02:11.96<br>02:11.80<br>−0.12	03:26.00<br>03:26.48<br>0.23	03:43.13<br>03:43.26<br>0.06	04:44.79<br>04:44.31<br>−0.17	07:20.67<br>07:21.08<br>0.09	12:39.36<br>12:38.16<br>−0.16	26:22.75<br>26:23.78<br>0.06	0.13	59:06.00<br>58:30.05<br>−1.01	2:05:42.00<br>2:02:46.52<br>−2.33
2002	05:45.46	413.68	0.38	6.10	0.104	0.055	02:11.96<br>02:11.80<br>−0.12	03:26.00<br>03:26.48<br>0.23	03:43.13<br>03:43.26<br>0.06	04:44.79<br>04:44.31<br>−0.17	07:20.67<br>07:21.08<br>0.09	12:39.36<br>12:38.16<br>−0.16	26:22.75<br>26:23.78<br>0.06	0.13	59:06.00<br>58:30.05<br>−1.01	2:05:37.80<br>2:02:46.52<br>−2.27

Year	t_c	v_m[m/min]	E_s	E_l	γ_{an}	γ_{ae}	1000	1500	1609.34	2000	3000	5000	10 000	av.err[%]	Halfmarathon	Marathon
2003	05:45.46	413.68	0.38	6.10	0.104	0.055	02:11.96<br>02:11.80<br>-0.12	03:26.00<br>03:26.48<br>0.23	03:43.13<br>03:43.26<br>0.06	04:44.79<br>04:44.31<br>-0.17	07:20.67<br>07:21.08<br>0.09	12:39.36<br>12:38.16<br>-0.16	26:22.75<br>26:23.78<br>0.06	0.13	59:06.00<br>58:30.05<br>-1.01	2:04:55.00<br>2:02:46.52<br>-1.71
2004	05:41.77	414.14	0.38	6.28	0.104	0.054	02:11.96<br>02:11.80<br>-0.12	03:26.00<br>03:26.48<br>0.23	03:43.13<br>03:43.26<br>0.06	04:44.79<br>04:44.31<br>-0.17	07:20.67<br>07:20.73<br>0.01	12:37.35<br>12:37.16<br>-0.02	26:20.31<br>26:20.47<br>0.01	0.09	59:06.00<br>58:19.50<br>-1.31	2:04:55.00<br>2:02:17.40<br>-2.10
2005	05:45.68	413.65	0.38	6.55	0.104	0.053	02:11.96<br>02:11.80<br>-0.12	03:26.00<br>03:26.48<br>0.23	03:43.13<br>03:43.26<br>0.06	04:44.79<br>04:44.31<br>-0.17	07:20.67<br>07:20.86<br>0.04	12:37.35<br>12:36.81<br>-0.07	26:17.53<br>26:17.99<br>0.03	0.10	59:05.00<br>58:09.46<br>-1.57	2:04:55.00<br>2:01:46.52<br>-2.51
2006	05:45.68	413.65	0.38	6.55	0.104	0.053	02:11.96<br>02:11.80<br>-0.12	03:26.00<br>03:26.48<br>0.23	03:43.13<br>03:43.26<br>0.06	04:44.79<br>04:44.31<br>-0.17	07:20.67<br>07:20.86<br>0.04	12:37.35<br>12:36.81<br>-0.07	26:17.53<br>26:17.99<br>0.03	0.10	58:55.00<br>58:09.46<br>-1.29	2:04:55.00<br>2:01:46.52<br>-2.51
2007	05:45.68	413.65	0.38	6.55	0.104	0.053	02:11.96<br>02:11.80<br>-0.12	03:26.00<br>03:26.48<br>0.23	03:43.13<br>03:43.26<br>0.06	04:44.79<br>04:44.31<br>-0.17	07:20.67<br>07:20.86<br>0.04	12:37.35<br>12:36.81<br>-0.07	26:17.53<br>26:17.99<br>0.03	0.10	58:33.00<br>58:09.46<br>-0.67	2:04:26.00<br>2:01:46.52<br>-2.14
2008	05:45.68	413.65	0.38	6.55	0.104	0.053	02:11.96<br>02:11.80<br>-0.12	03:26.00<br>03:26.48<br>0.23	03:43.13<br>03:43.26<br>0.06	04:44.79<br>04:44.31<br>-0.17	07:20.67<br>07:20.86<br>0.04	12:37.35<br>12:36.81<br>-0.07	26:17.53<br>26:17.99<br>0.03	0.10	58:33.00<br>58:09.46<br>-0.67	2:03:58.20<br>2:01:46.52<br>-1.77
2009	05:45.68	413.65	0.38	6.55	0.104	0.053	02:11.96<br>02:11.80<br>-0.12	03:26.00<br>03:26.48<br>0.23	03:43.13<br>03:43.26<br>0.06	04:44.79<br>04:44.31<br>-0.17	07:20.67<br>07:20.86<br>0.04	12:37.35<br>12:36.81<br>-0.07	26:17.53<br>26:17.99<br>0.03	0.10	58:33.00<br>58:09.46<br>-0.67	2:03:58.20<br>2:01:46.52<br>-1.77
2010	05:45.68	413.65	0.38	6.55	0.104	0.053	02:11.96<br>02:11.80<br>-0.12	03:26.00<br>03:26.48<br>0.23	03:43.13<br>03:43.26<br>0.06	04:44.79<br>04:44.31<br>-0.17	07:20.67<br>07:20.86<br>0.04	12:37.35<br>12:36.81<br>-0.07	26:17.53<br>26:17.99<br>0.03	0.10	58:23.00<br>58:09.46<br>-0.39	2:03:58.20<br>2:01:46.52<br>-1.77
2011	05:45.68	413.65	0.38	6.55	0.104	0.053	02:11.96<br>02:11.80<br>-0.12	03:26.00<br>03:26.48<br>0.23	03:43.13<br>03:43.26<br>0.06	04:44.79<br>04:44.31<br>-0.17	07:20.67<br>07:20.86<br>0.04	12:37.35<br>12:36.81<br>-0.07	26:17.53<br>26:17.99<br>0.03	0.10	58:23.00<br>58:09.46<br>-0.39	2:03:38.00<br>2:01:46.52<br>-1.50
2012	05:45.68	413.65	0.38	6.55	0.104	0.053	02:11.96<br>02:11.80<br>-0.12	03:26.00<br>03:26.48<br>0.23	03:43.13<br>03:43.26<br>0.06	04:44.79<br>04:44.31<br>-0.17	07:20.67<br>07:20.86<br>0.04	12:37.35<br>12:36.81<br>-0.07	26:17.53<br>26:17.99<br>0.03	0.10	58:23.00<br>58:09.46<br>-0.39	2:03:38.00<br>2:01:46.52<br>-1.50
2013	05:45.68	413.65	0.38	6.55	0.104	0.053	02:11.96<br>02:11.80<br>-0.12	03:26.00<br>03:26.48<br>0.23	03:43.13<br>03:43.26<br>0.06	04:44.79<br>04:44.31<br>-0.17	07:20.67<br>07:20.86<br>0.04	12:37.35<br>12:36.81<br>-0.07	26:17.53<br>26:17.99<br>0.03	0.10	58:23.00<br>58:09.46<br>-0.39	2:03:22.20<br>2:01:46.52<br>-1.29
2014	05:45.68	413.65	0.38	6.55	0.104	0.053	02:11.96<br>02:11.80<br>-0.12	03:26.00<br>03:26.48<br>0.23	03:43.13<br>03:43.26<br>0.06	04:44.79<br>04:44.31<br>-0.17	07:20.67<br>07:20.86<br>0.04	12:37.35<br>12:36.81<br>-0.07	26:17.53<br>26:17.99<br>0.03	0.10	58:23.00<br>58:09.46<br>-0.39	2:02:56.40<br>2:01:46.52<br>-0.95
2015	05:45.68	413.65	0.38	6.55	0.104	0.053	02:11.96<br>02:11.80<br>-0.12	03:26.00<br>03:26.48<br>0.23	03:43.13<br>03:43.26<br>0.06	04:44.79<br>04:44.31<br>-0.17	07:20.67<br>07:20.86<br>0.04	12:37.35<br>12:36.81<br>-0.07	26:17.53<br>26:17.99<br>0.03	0.10	58:23.00<br>58:09.46<br>-0.39	2:02:56.40<br>2:01:46.52<br>-0.95
2016	05:45.68	413.65	0.38	6.55	0.104	0.053	02:11.96<br>02:11.80<br>-0.12	03:26.00<br>03:26.48<br>0.23	03:43.13<br>03:43.26<br>0.06	04:44.79<br>04:44.31<br>-0.17	07:20.67<br>07:20.86<br>0.04	12:37.35<br>12:36.81<br>-0.07	26:17.53<br>26:17.99<br>0.03	0.10	58:23.00<br>58:09.46<br>-0.39	2:02:56.40<br>2:01:46.52<br>-0.95
2017	05:45.68	413.65	0.38	6.55	0.104	0.053	02:11.96<br>02:11.80<br>-0.12	03:26.00<br>03:26.48<br>0.23	03:43.13<br>03:43.26<br>0.06	04:44.79<br>04:44.31<br>-0.17	07:20.67<br>07:20.86<br>0.04	12:37.35<br>12:36.81<br>-0.07	26:17.53<br>26:17.99<br>0.03	0.10	58:23.00<br>58:09.46<br>-0.39	2:02:56.40<br>2:01:46.52<br>-0.95
2018	05:45.68	413.65	0.38	6.55	0.104	0.053	02:11.96<br>02:11.80<br>-0.12	03:26.00<br>03:26.48<br>0.23	03:43.13<br>03:43.26<br>0.06	04:44.79<br>04:44.31<br>-0.17	07:20.67<br>07:20.86<br>0.04	12:37.35<br>12:36.81<br>-0.07	26:17.53<br>26:17.99<br>0.03	0.10	58:18.00<br>58:09.46<br>-0.24	2:01:39.00<br>2:01:46.52<br>0.10
2019	05:45.68	413.65	0.38	6.55	0.104	0.053	02:11.96<br>02:11.80<br>-0.12	03:26.00<br>03:26.48<br>0.23	03:43.13<br>03:43.26<br>0.06	04:44.79<br>04:44.31<br>-0.17	07:20.67<br>07:20.86<br>0.04	12:37.35<br>12:36.81<br>-0.07	26:17.53<br>26:17.99<br>0.03	0.10	58:01.00<br>58:09.46<br>0.24	2:01:39.00<br>2:01:46.52<br>0.10

Year	t_c	v_m [m/min]	E_s	E_l	γ_{an}	γ_{ae}	1000	1500	1609.34	2000	3000	5000	10 000	av.err [%]	Halfmarathon	Marathon
2020	05:47.74	413.39	0.38	7.24	0.104	0.051	02:11.96<br>02:11.80<br>−0.12	03:26.00<br>03:26.48<br>0.23	03:43.13<br>03:43.26<br>0.06	04:44.79<br>04:44.31<br>−0.17	07:20.67<br>07:20.69<br>0.01	12:35.36<br>12:35.29<br>−0.01	26:11.00<br>26:11.06<br>0.00	0.09	57:32.00<br>57:44.30<br>0.36	2:01:39.00<br>2:00:32.80<br>−0.91
2021	05:47.74	413.39	0.38	7.24	0.104	0.051	02:11.96<br>02:11.80<br>−0.12	03:26.00<br>03:26.48<br>0.23	03:43.13<br>03:43.26<br>0.06	04:44.79<br>04:44.31<br>−0.17	07:20.67<br>07:20.69<br>0.01	12:35.36<br>12:35.29<br>−0.01	26:11.00<br>26:11.06<br>0.00	0.09	57:31.00<br>57:44.30<br>0.39	2:01:39.00<br>2:00:32.80<br>−0.91
2022	05:47.74	413.39	0.38	7.24	0.104	0.051	02:11.96<br>02:11.80<br>−0.12	03:26.00<br>03:26.48<br>0.23	03:43.13<br>03:43.26<br>0.06	04:44.79<br>04:44.31<br>−0.17	07:20.67<br>07:20.69<br>0.01	12:35.36<br>12:35.29<br>−0.01	26:11.00<br>26:11.06<br>0.00	0.09	57:31.00<br>57:44.30<br>0.39	2:01:09.00<br>2:00:32.80<br>−0.50
2023	06:23.09	411.37	0.36	7.17	0.099	0.051	02:11.96<br>02:11.97<br>0.01	03:26.00<br>03:26.17<br>0.08	03:43.13<br>03:42.81<br>−0.14	04:43.13<br>04:43.27<br>0.05	07:20.67<br>07:20.69<br>0.01	12:35.36<br>12:35.29<br>−0.01	26:11.00<br>26:11.06<br>0.00	0.04	57:31.00<br>57:44.30<br>0.39	2:00:35.00<br>2:00:32.80<br>−0.03

World Records Women

Year	t_c	v_m[m/min]	E_s	E_l	γ_{an}	γ_{ae}	1000	1500	1609.34	2000	3000	5000	10 000	av.err[%]	Halfmarathon	Marathon
1984	07:40.08	356.87	0.34	3.90	0.093	0.073	02:33.80	03:52.47	04:17.44	05:28.72	08:22.62	14:58.89	31:00.00	1.13	1:08:34.00	2:22:43.00
							02:32.46	03:57.58	04:16.63	05:25.79	08:28.08	14:42.89	31:14.64		1:10:38.07	2:31:23.71
							-0.87	2.20	-0.31	-0.89	1.09	-1.78	0.79		3.02	6.08
1985	06:25.29	363.14	0.33	3.93	0.091	0.073	02:33.80	03:52.47	04:16.71	05:28.72	08:22.62	14:48.07	30:59.42	0.88	1:08:34.00	2:21:06.00
							02:32.40	03:57.41	04:16.43	05:25.47	08:25.71	14:39.06	31:07.42		1:10:24.32	2:31:00.30
							-0.91	2.12	-0.11	-0.99	0.62	-1.02	0.43		2.68	7.02
1986	07:07.94	359.72	0.34	5.63	0.091	0.058	02:33.80	03:52.47	04:16.71	05:28.69	08:22.62	14:37.33	30:13.74	0.84	1:08:34.00	2:21:06.00
							02:32.41	03:57.40	04:16.42	05:25.45	08:25.25	14:29.67	30:20.49		1:07:24.67	2:21:50.32
							-0.91	2.12	-0.11	-0.99	0.52	-0.87	0.37		-1.69	0.52
1987	07:07.94	359.72	0.34	5.63	0.091	0.058	02:33.80	03:52.47	04:16.71	05:28.69	08:22.62	14:37.33	30:13.74	0.84	1:06:40.00	2:21:06.00
							02:32.41	03:57.40	04:16.42	05:25.45	08:25.25	14:29.67	30:20.49		1:07:24.67	2:21:50.32
							-0.91	2.12	-0.11	-0.99	0.52	-0.87	0.37		1.12	0.52
1988	07:07.94	359.72	0.34	5.63	0.091	0.058	02:33.80	03:52.47	04:16.71	05:28.69	08:22.62	14:37.33	30:13.74	0.84	1:06:40.00	2:21:06.00
							02:32.41	03:57.40	04:16.42	05:25.45	08:25.25	14:29.67	30:20.49		1:07:24.67	2:21:50.32
							-0.91	2.12	-0.11	-0.99	0.52	-0.87	0.37		1.12	0.52
1989	07:30.41	358.65	0.33	5.60	0.091	0.058	02:33.80	03:52.47	04:15.61	05:28.69	08:22.62	14:37.33	30:13.74	0.87	1:06:40.00	2:21:06.00
							02:32.32	03:57.15	04:16.12	05:24.97	08:25.25	14:29.67	30:20.49		1:07:24.67	2:21:50.32
							-0.96	2.01	0.20	-1.13	0.52	-0.87	0.37		1.12	0.52
1990	06:03.72	363.10	0.42	5.72	0.117	0.057	02:30.67	03:52.47	04:15.61	05:28.69	08:22.62	14:37.33	30:13.74	0.67	1:06:40.00	2:21:06.00
							02:29.72	03:55.93	04:15.38	05:26.35	08:25.25	14:29.67	30:20.49		1:07:24.67	2:21:50.32
							-0.63	1.49	-0.09	-0.71	0.52	-0.87	0.37		1.12	0.52
1991	06:03.72	363.10	0.42	5.72	0.117	0.057	02:30.67	03:52.47	04:15.61	05:28.69	08:22.62	14:37.33	30:13.74	0.67	1:06:40.00	2:21:06.00
							02:29.72	03:55.93	04:15.38	05:26.35	08:25.25	14:29.67	30:20.49		1:07:24.67	2:21:50.32
							-0.63	1.49	-0.09	-0.71	0.52	-0.87	0.37		1.12	0.52
1992	06:03.72	363.10	0.42	5.72	0.117	0.057	02:30.67	03:52.47	04:15.61	05:28.69	08:22.62	14:37.33	30:13.74	0.67	1:06:40.00	2:21:06.00
							02:29.72	03:55.93	04:15.38	05:26.35	08:25.25	14:29.67	30:20.49		1:07:24.67	2:21:50.32
							-0.63	1.49	-0.09	-0.71	0.52	-0.87	0.37		1.12	0.52
1993	05:28.69	369.65	0.40	6.28	0.108	0.054	02:30.67	03:50.46	04:15.61	05:28.69	08:06.11	14:37.33	29:31.78	1.49	1:06:40.00	2:21:06.00
							02:29.63	03:54.97	04:14.18	05:24.14	08:18.22	14:16.20	29:48.01		1:06:01.22	2:18:30.19
							-0.69	1.96	-0.56	-1.38	2.49	-2.41	0.92		-0.97	-1.84
1994	05:25.36	371.56	0.37	5.67	0.100	0.058	02:30.67	03:50.46	04:15.61	05:25.36	08:06.11	14:37.33	29:31.78	1.38	1:06:40.00	2:21:06.00
							02:29.88	03:54.55	04:13.56	05:22.70	08:16.54	14:15.00	29:50.75		1:06:21.24	2:19:42.63
							-0.53	1.78	-0.80	-0.82	2.15	-2.55	1.07		-0.47	-0.98
1995	05:25.36	371.14	0.40	5.85	0.111	0.057	02:29.34	03:50.46	04:15.61	05:25.36	08:06.11	14:36.45	29:31.78	1.31	1:06:40.00	2:21:06.00
							02:28.80	03:53.97	04:13.15	05:23.08	08:16.90	14:15.08	29:49.23		1:06:13.37	2:19:16.26
							-0.36	1.52	-0.96	-0.70	2.22	-2.44	0.99		-0.67	-1.30
1996	05:25.36	372.02	0.41	5.57	0.112	0.058	02:28.98	03:50.46	04:12.56	05:25.36	08:06.11	14:36.45	29:31.78	1.19	1:06:40.00	2:21:06.00
							02:28.22	03:53.22	04:12.37	05:22.22	08:16.03	14:14.46	29:50.68		1:06:23.88	2:19:54.34
							-0.51	1.20	-0.07	-0.97	2.04	-2.51	1.07		-0.40	-0.85
1997	05:25.36	372.42	0.40	5.81	0.110	0.057	02:28.98	03:50.46	04:12.56	05:25.36	08:06.11	14:28.09	29:31.78	1.03	1:06:40.00	2:21:06.00
							02:28.29	03:53.11	04:12.21	05:21.83	08:15.15	14:12.19	29:43.61		1:06:01.99	2:18:54.73
							-0.47	1.15	-0.14	-1.08	1.86	-1.83	0.67		-0.95	-1.55
1998	05:25.36	372.42	0.40	5.81	0.110	0.057	02:28.98	03:50.46	04:12.56	05:25.36	08:06.11	14:28.09	29:31.78	1.03	1:06:40.00	2:20:47.00
							02:28.29	03:53.11	04:12.21	05:21.83	08:15.15	14:12.19	29:43.61		1:06:01.99	2:18:54.73
							-0.47	1.15	-0.14	-1.08	1.86	-1.83	0.67		-0.95	-1.33
1999	05:25.36	372.42	0.40	5.81	0.110	0.057	02:28.98	03:50.46	04:12.56	05:25.36	08:06.11	14:28.09	29:31.78	1.03	1:06:40.00	2:20:43.00
							02:28.29	03:53.11	04:12.21	05:21.83	08:15.15	14:12.19	29:43.61		1:06:01.99	2:18:54.73
							-0.47	1.15	-0.14	-1.08	1.86	-1.83	0.67		-0.95	-1.28
2000	05:25.36	372.42	0.40	5.81	0.110	0.057	02:28.98	03:50.46	04:12.56	05:25.36	08:06.11	14:28.09	29:31.78	1.03	1:06:40.00	2:20:43.00
							02:28.29	03:53.11	04:12.21	05:21.83	08:15.15	14:12.19	29:43.61		1:06:01.99	2:18:54.73
							-0.47	1.15	-0.14	-1.08	1.86	-1.83	0.67		-0.95	-1.28

Year	t_c	v_m[m/min]	E_s	E_l	γ_{an}	γ_{ae}	1000	1500	1609.34	2000	3000	5000	10 000	av.err[%]	Halfmarathon	Marathon
2001	05:25.36	372.42	0.40	5.81	0.110	0.057	02:28.98<br>02:28.29<br>−0.47	03:50.46<br>03:53.11<br>1.15	04:12.56<br>04:12.21<br>−0.14	05:25.36<br>05:21.83<br>−1.08	08:06.11<br>08:15.15<br>1.86	14:28.09<br>14:12.19<br>−1.83	29:31.78<br>29:43.61<br>0.67	1.03	1:05:44.00<br>1:06:01.99<br>0.46	2:18:47.00<br>2:18:54.73<br>0.09
2002	05:25.36	372.42	0.40	5.81	0.110	0.057	02:28.98<br>02:28.29<br>−0.47	03:50.46<br>03:53.11<br>1.15	04:12.56<br>04:12.21<br>−0.14	05:25.36<br>05:21.83<br>−1.08	08:06.11<br>08:15.15<br>1.86	14:28.09<br>14:12.19<br>−1.83	29:31.78<br>29:43.61<br>0.67	1.03	1:05:44.00<br>1:06:01.99<br>0.46	2:17:17.80<br>2:18:54.73<br>1.18
2003	05:25.36	372.42	0.40	5.81	0.110	0.057	02:28.98<br>02:28.29<br>−0.47	03:50.46<br>03:53.11<br>1.15	04:12.56<br>04:12.21<br>−0.14	05:25.36<br>05:21.83<br>−1.08	08:06.11<br>08:15.15<br>1.86	14:28.09<br>14:12.19<br>−1.83	29:31.78<br>29:43.61<br>0.67	1.03	1:05:39.60<br>1:06:01.99<br>0.57	2:15:24.60<br>2:18:54.73<br>2.59
2004	05:25.36	372.59	0.40	5.91	0.109	0.056	02:28.98<br>02:28.31<br>−0.45	03:50.46<br>03:53.06<br>1.13	04:12.56<br>04:12.14<br>−0.17	05:25.36<br>05:21.67<br>−1.13	08:06.11<br>08:14.78<br>1.78	14:24.68<br>14:11.28<br>−1.55	29:31.78<br>29:40.80<br>0.51	0.96	1:05:39.60<br>1:05:53.35<br>0.35	2:15:24.60<br>2:18:31.32<br>2.30
2005	05:25.36	372.59	0.40	5.91	0.109	0.056	02:28.98<br>02:28.31<br>−0.45	03:50.46<br>03:53.06<br>1.13	04:12.56<br>04:12.14<br>−0.17	05:25.36<br>05:21.67<br>−1.13	08:06.11<br>08:14.78<br>1.78	14:24.68<br>14:11.28<br>−1.55	29:31.78<br>29:40.80<br>0.51	0.96	1:05:39.60<br>1:05:53.35<br>0.35	2:15:24.60<br>2:18:31.32<br>2.30
2006	05:25.36	372.60	0.40	5.91	0.109	0.056	02:28.98<br>02:28.31<br>−0.45	03:50.46<br>03:53.06<br>1.13	04:12.56<br>04:12.14<br>−0.17	05:25.36<br>05:21.66<br>−1.14	08:06.11<br>08:14.77<br>1.78	14:24.53<br>14:11.24<br>−1.54	29:31.78<br>29:40.68<br>0.50	0.96	1:05:39.60<br>1:05:52.97<br>0.34	2:15:24.60<br>2:18:30.30<br>2.29
2007	05:25.36	373.00	0.39	6.15	0.107	0.055	02:28.98<br>02:28.38<br>−0.40	03:50.46<br>03:52.95<br>1.08	04:12.56<br>04:11.98<br>−0.23	05:25.36<br>05:21.28<br>−1.25	08:06.11<br>08:13.93<br>1.61	14:16.63<br>14:09.15<br>−0.87	29:31.78<br>29:34.32<br>0.14	0.80	1:05:39.60<br>1:05:33.58<br>−0.15	2:15:24.60<br>2:17:38.07<br>1.64
2008	05:25.36	373.29	0.39	6.31	0.106	0.054	02:28.98<br>02:28.42<br>−0.37	03:50.46<br>03:52.87<br>1.05	04:12.56<br>04:11.86<br>−0.28	05:25.36<br>05:21.01<br>−1.34	08:06.11<br>08:13.35<br>1.49	14:11.15<br>14:07.72<br>−0.40	29:31.78<br>29:30.03<br>−0.10	0.72	1:05:39.60<br>1:05:20.64<br>−0.48	2:15:24.60<br>2:17:03.41<br>1.22
2009	05:25.36	373.29	0.39	6.31	0.106	0.054	02:28.98<br>02:28.42<br>−0.37	03:50.46<br>03:52.87<br>1.05	04:12.56<br>04:11.86<br>−0.28	05:25.36<br>05:21.01<br>−1.34	08:06.11<br>08:13.35<br>1.49	14:11.15<br>14:07.72<br>−0.40	29:31.78<br>29:30.03<br>−0.10	0.72	1:05:39.60<br>1:05:20.64<br>−0.48	2:15:24.60<br>2:17:03.41<br>1.22
2010	05:25.36	373.29	0.39	6.31	0.106	0.054	02:28.98<br>02:28.42<br>−0.37	03:50.46<br>03:52.87<br>1.05	04:12.56<br>04:11.86<br>−0.28	05:25.36<br>05:21.01<br>−1.34	08:06.11<br>08:13.35<br>1.49	14:11.15<br>14:07.72<br>−0.40	29:31.78<br>29:30.03<br>−0.10	0.72	1:05:39.60<br>1:05:20.64<br>−0.48	2:15:24.60<br>2:17:03.41<br>1.22
2011	05:25.36	373.29	0.39	6.31	0.106	0.054	02:28.98<br>02:28.42<br>−0.37	03:50.46<br>03:52.87<br>1.05	04:12.56<br>04:11.86<br>−0.28	05:25.36<br>05:21.01<br>−1.34	08:06.11<br>08:13.35<br>1.49	14:11.15<br>14:07.72<br>−0.40	29:31.78<br>29:30.03<br>−0.10	0.72	1:05:39.60<br>1:05:20.64<br>−0.48	2:15:24.60<br>2:17:03.41<br>1.22
2012	05:25.36	373.29	0.39	6.31	0.106	0.054	02:28.98<br>02:28.42<br>−0.37	03:50.46<br>03:52.87<br>1.05	04:12.56<br>04:11.86<br>−0.28	05:25.36<br>05:21.01<br>−1.34	08:06.11<br>08:13.35<br>1.49	14:11.15<br>14:07.72<br>−0.40	29:31.78<br>29:30.03<br>−0.10	0.72	1:05:39.60<br>1:05:20.64<br>−0.48	2:15:24.60<br>2:17:03.41<br>1.22
2013	05:25.36	373.29	0.39	6.31	0.106	0.054	02:28.98<br>02:28.42<br>−0.37	03:50.46<br>03:52.87<br>1.05	04:12.56<br>04:11.86<br>−0.28	05:25.36<br>05:21.01<br>−1.34	08:06.11<br>08:13.35<br>1.49	14:11.15<br>14:07.72<br>−0.40	29:31.78<br>29:30.03<br>−0.10	0.72	1:05:39.60<br>1:05:20.64<br>−0.48	2:15:24.60<br>2:17:03.41<br>1.22
2014	05:25.36	373.29	0.39	6.31	0.106	0.054	02:28.98<br>02:28.42<br>−0.37	03:50.46<br>03:52.87<br>1.05	04:12.56<br>04:11.86<br>−0.28	05:25.36<br>05:21.01<br>−1.34	08:06.11<br>08:13.35<br>1.49	14:11.15<br>14:07.72<br>−0.40	29:31.78<br>29:30.03<br>−0.10	0.72	1:05:12.00<br>1:05:20.64<br>0.22	2:15:24.60<br>2:17:03.41<br>1.22
2015	05:25.36	373.37	0.39	6.28	0.106	0.054	02:28.98<br>02:28.35<br>−0.42	03:50.07<br>03:52.79<br>1.18	04:12.56<br>04:11.78<br>−0.31	05:25.36<br>05:20.93<br>−1.36	08:06.11<br>08:13.26<br>1.47	14:11.15<br>14:07.66<br>−0.41	29:31.78<br>29:30.17<br>−0.09	0.75	1:05:09.00<br>1:05:21.63<br>0.32	2:15:24.60<br>2:17:07.00<br>1.26
2016	05:25.36	372.98	0.40	7.21	0.108	0.051	02:28.98<br>02:28.29<br>−0.47	03:50.07<br>03:52.89<br>1.23	04:12.56<br>04:11.93<br>−0.25	05:25.36<br>05:21.30<br>−1.25	08:06.11<br>08:12.97<br>1.41	14:11.15<br>14:05.18<br>−0.70	29:17.45<br>29:18.93<br>0.08	0.77	1:05:09.00<br>1:04:40.89<br>−0.72	2:15:24.60<br>2:15:07.52<br>−0.21
2017	05:23.75	373.91	0.38	6.80	0.104	0.052	02:28.98<br>02:28.41<br>−0.39	03:50.07<br>03:52.69<br>1.14	04:12.56<br>04:11.64<br>−0.36	05:23.75<br>05:20.61<br>−0.97	08:06.11<br>08:12.16<br>1.24	14:11.15<br>14:04.60<br>−0.77	29:17.45<br>29:20.21<br>0.16	0.72	1:04:51.00<br>1:04:50.19<br>−0.02	2:15:24.60<br>2:15:40.86<br>0.20

Year	t_c	v_m[m/min]	E_s	E_l	γ_{an}	γ_{ae}	1000	1500	1609.34	2000	3000	5000	10 000	av.err[%]	Halfmarathon	Marathon
2018	05:23.75	373.91	0.38	6.80	0.104	0.052	02:28.98<br>02:28.41<br>−0.39	03:50.07<br>03:52.69<br>1.14	04:12.56<br>04:11.64<br>−0.36	05:23.75<br>05:20.61<br>−0.97	08:06.11<br>08:12.16<br>1.24	14:11.15<br>14:04.60<br>−0.77	29:17.45<br>29:20.21<br>0.16	0.72	1:04:51.00<br>1:04:50.19<br>−0.02	2:15:24.60<br>2:15:40.86<br>0.20
2019	05:23.75	373.98	0.38	6.76	0.104	0.052	02:28.98<br>02:28.38<br>−0.40	03:50.07<br>03:52.65<br>1.12	04:12.33<br>04:11.59<br>−0.29	05:23.75<br>05:20.54<br>−0.99	08:06.11<br>08:12.09<br>1.23	14:11.15<br>14:04.55<br>−0.78	29:17.45<br>29:20.32<br>0.16	0.71	1:04:51.00<br>1:04:51.04<br>0.00	2:14:04.00<br>2:15:43.91<br>1.24
2020	05:23.75	374.22	0.38	6.93	0.103	0.052	02:28.98<br>02:28.42<br>−0.37	03:50.07<br>03:52.58<br>1.09	04:12.33<br>04:11.50<br>−0.33	05:23.75<br>05:20.32<br>−1.06	08:06.11<br>08:11.61<br>1.13	14:06.62<br>14:03.37<br>−0.38	29:17.45<br>29:16.79<br>−0.04	0.63	1:04:31.00<br>1:04:40.42<br>0.24	2:14:04.00<br>2:15:15.63<br>0.89
2021	05:21.56	375.02	0.37	7.58	0.100	0.049	02:28.98<br>02:28.51<br>−0.32	03:50.07<br>03:52.44<br>1.03	04:12.33<br>04:11.28<br>−0.42	05:21.56<br>05:19.80<br>−0.55	08:06.11<br>08:10.17<br>0.84	14:06.62<br>13:59.75<br>−0.81	29:01.03<br>29:05.70<br>0.27	0.60	1:02:52.00<br>1:04:06.71<br>1.98	2:14:04.00<br>2:13:45.50<br>−0.23
2022	05:21.56	375.02	0.37	7.58	0.100	0.049	02:28.98<br>02:28.51<br>−0.32	03:50.07<br>03:52.44<br>1.03	04:12.33<br>04:11.28<br>−0.42	05:21.56<br>05:19.80<br>−0.55	08:06.11<br>08:10.17<br>0.84	14:06.62<br>13:59.75<br>−0.81	29:01.03<br>29:05.70<br>0.27	0.60	1:02:52.00<br>1:04:06.71<br>1.98	2:14:04.00<br>2:13:45.50<br>−0.23
2023	05:21.56	377.10	0.36	6.93	0.098	0.052	02:28.98<br>02:27.90<br>−0.72	03:49.11<br>03:51.22<br>0.92	04:07.64<br>04:09.92<br>0.92	05:21.56<br>05:17.86<br>−1.15	08:06.11<br>08:07.84<br>0.36	14:00.21<br>13:56.92<br>−0.39	29:01.03<br>29:03.39<br>0.14	0.66	1:02:52.00<br>1:04:10.90<br>2.09	2:11:53.00<br>2:14:14.08<br>1.78
